# Controlling Molecular Aggregation-Induced Emission by Controlled Polymerization

**DOI:** 10.3390/molecules26206267

**Published:** 2021-10-16

**Authors:** Yinyin Bao

**Affiliations:** Department of Chemistry and Applied Biosciences, Institute of Pharmaceutical Sciences, ETH Zurich, Vladimir-Prelog-Weg 1-5/10, 8093 Zurich, Switzerland; yinyin.bao@pharma.ethz.ch or baoyinyin@mail.ustc.edu.cn

**Keywords:** aggregation-induced emission, single fluorophore, ring-opening polymerization, controlled radical polymerization, fluorescent polymer

## Abstract

In last twenty years, the significant development of AIE materials has been witnessed. A number of small molecules, polymers and composites with AIE activity have been synthesized, with some of these exhibiting great potential in optoelectronics and biomedical applications. Compared to AIE small molecules, macromolecular systems—especially well-defined AIE polymers—have been studied relatively less. Controlled polymerization methods provide the efficient synthesis of well-defined AIE polymers with varied monomers, tunable chain lengths and narrow dispersity. In particular, the preparation of single-fluorophore polymers through AIE molecule-initiated polymerization enables the systematic investigation of the structure–property relationships of AIE polymeric systems. Here, the main polymerization techniques involved in these polymers are summarized and the key parameters that affect their photophysical properties are analyzed. The author endeavored to collect meaningful information from the descriptions of AIE polymer systems in the literature, to find connections by comparing different representative examples, and hopes eventually to provide a set of general guidelines for AIE polymer design, along with personal perspectives on the direction of future research.

## 1. Introduction

The manipulation of the fluorescence intensity and emission color of molecular fluorophores, especially in their solid or aggregated states, is of fundamental importance for both scientific research and real-world applications [1,2,3,4]. The most common strategy is the derivatization of certain fluorophore scaffolds with different side groups. Usually based on intramolecular charge transfer (ICT), this method can be used to efficiently tune the fluorescence quantum yield and emission wavelength of the resulting molecules [5]. Alternatively, the incorporation of one or more molecular fluorophores onto polymeric platforms can also enhance the emission and can sometimes produce multicolor fluorescence [6,7,8]. Nevertheless, these systems either require tedious organic syntheses or possess complicated chemical compositions. Recently, single-fluorophore-based emissive materials have shown the potential to allow fluorescence manipulation in a rather simple manner, especially when aggregation-induced emission (AIE)-active fluorophores are used [9,10,11].

Coined by Tang in 2001, the concept of AIE has undergone enormous developments, with wide applications, such as optoelectronic devices, fluorescence sensing, bioimaging, diagnostics, cancer therapy and process visualization [12,13,14,15,16,17]. In the 20 years of AIE advancement, a great number of functional organic molecules and polymers have been designed and synthesized [18,19,20], which has significantly enriched molecular light-emitting systems. Generally, the structure–property relationships of AIE-active molecules have been extensively investigated on the basis of the restriction of motion mechanism [21] and the intramolecular charge transfer process of the π-conjugation structure [5]. As a result, the AIE properties of small molecules can be manipulated through sophisticated structural design and organic synthesis. However, the nature of AIE-active polymeric systems, due to the complex effect of polymer chains on both their aggregate state and charge transfer, remains less well understood, especially for those based on non-π-conjugated structures. Polymerization methods are often used only as a tool to offer AIE fluorophore soft matrixes or biocompatible carriers [22], and thus the influence on the emission properties brought about by the attachment of polymer chains itself, is less frequently taken into consideration.

In recent years, a series of single-fluorophore-based AIE polymers synthesized through controlled polymerization have been reported, which hold well-defined polymeric structures, bearing only one fluorophore in each polymer chain [23,24]. This unique chemical structure offers a convenient way to investigate the photophysical properties of AIE polymers. Despite this face, a comprehensive analysis of their structure–property relationships has not been disclosed. In this perspective article, the author endeavored to collect meaningful information from the single-fluorophore AIE polymer systems described in the literature, and find connections by comparing different representative examples, which might be of interest to researchers in the fields of both light-emitting materials and polymer science. Here, the focus is on the structure–property relationships of well-defined single-fluorophore-based AIE polymers; therefore, π-conjugated polymers, which represent another type of AIE system, are not included.

## 2. AIE Fluorophore-Initiated Controlled Polymerization

**Figure 1:**
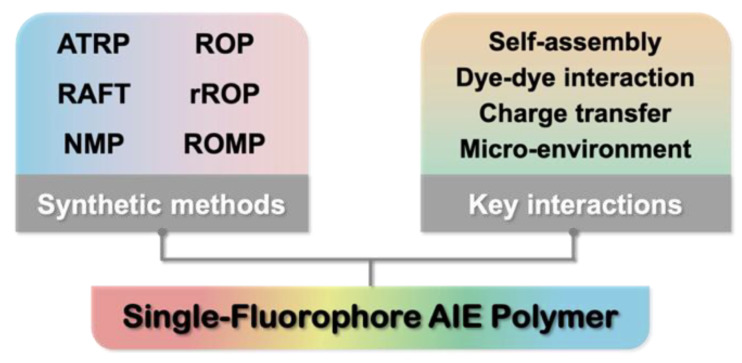
Schematic illustration of the main synthetic methods and key interactions involved in single-fluorophore AIE polymers.

Controlled polymerizations, especially controlled radical polymerizations or reversible deactivation radical polymerizations, have shown powerful abilities in the preparation of various functional polymeric materials, including light-emitting polymers [25,26]. In particular, functionalized AIE fluorophores have been used as initiators or monomers to prepare well-defined fluorescent polymers, owing to their flexible structural design and the convenient processing. Polymerization methods include atom transfer radical polymerization (ATRP) [27], reversible addition-fragmentation chain transfer (RAFT) polymerization [28], nitroxide-mediated polymerization (NMP) [29], ring-opening polymerization (ROP) [30] and others. In general, one highly convenient and efficient approach to synthesize well-defined fluorescent polymers is to functionalize the fluorophore with an initiating group or transfer agent for controlled polymerization [31,32,33,34]. In this way, each polymer chain only bears a single fluorophore so that the impact of the polymer chains or matrix can be more quantitatively evaluated, excluding the interference resulting from multiple interactions. Clearly, this principle also applies to AIE-active polymers, as indicated by versatile AIE fluorophore-initiated controlled polymerization. In addition to controlled radical polymerization and conventional ROP, ring-opening metathesis polymerization (ROMP) [35] and radical ring-opening polymerization (rROP) [36] have also emerged as efficient tools for AIE polymer synthesis. Thanks to these powerful polymerization techniques, the key parameters that can affect the photophysical properties of AIE polymers have been gradually revealed, including micro-environment, dye–dye interaction, self-assembly and charge transfer (Figure 1).

## 3. Single-Fluorophore AIE Polymers Prepared by Controlled Radical Polymerization 

**Figure 2:**
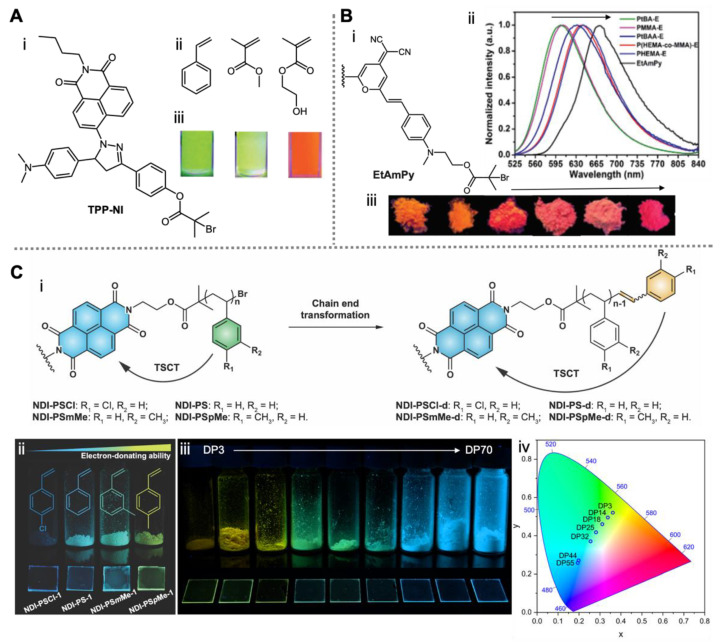
Single-fluorophore AIE polymers prepared by ATRP. (**A**) Structure of a naphthalimide-based AIE initiator (i) and the corresponding monomers (ii), as well as the photographs of the resulting polymers under UV irradiation in their aggregate state (iii). Reproduced with permission from [40], copyright 2012, Royal Society of Chemistry. (**B**) Structure of an ICT initiator (i), the fluorescence spectra of the various resulting polymers in their aggregated state (ii) and the photographs of the polymer powders under UV irradiation (iii). Reproduced with permission from [44], copyright 2018, Royal Society of Chemistry. (**C**) Structure of **NDI**-based TSCT polymers before and after end group transformation (i), photographs of the polymers using different styrenic monomers (ii) or using styrene with different chain lengths under UV irradiation (iii) and the corresponding CIE coordinates (iv). Reproduced with permission from [47], open access, American Association for the Advancement of Science.

Because it is extremely easy to introduce the halogen radical-generating moiety to molecules with hydroxyl or amine groups using commercial acyl halide reagents, ATRP has been the most reported method to design single-fluorophore polymers [37,38,39]. In 2012, Xu, Lu and co-workers used a naphthalimide-based AIE active initiator (TPP-NI) to prepare a series of well-defined fluorescent polymers by means of ATRP, including polystyrene (PS), poly(methyl methacrylate) (PMMA) and poly (2-hydroxyethyl methacrylate) (PHEMA) [40]. They found that the fluorescence quantum yield of AIE-active polystyrene in its aggregated state can be enhanced by increasing the polymer chain length, which is attributed to the higher degree of molecular motion restriction in longer polymer chains [41]. Similar phenomena have been observed in other systems [42,43]. Interestingly, the polymer film of PHEMA showed a distinct emission color compared to PS and PMMA (Figure 2A). The former maintained the emission wavelength of the molecular fluorophore in its solid state (~600 nm), whereas the latter two exhibited significant blue-shifts (~560 nm). This may provide opportunities to manipulate the emission color of AIE fluorophores simply by selecting monomers with different polarity.

Later on, the same group synthesized another AIE-active initiator (EtAmPy) with an intramolecular charge transfer (ICT) effect, which was used to synthesize single-fluorophore-based polymers including PMMA, poly(tert-butyl methacrylate) (P*t*BA), PHEMA and their derivatives [44]. It was found that the emission peak can shift from ~640 nm to ~600 nm upon reducing the polarity of the polymers, whereas the AIE molecule showed maximum emission at ~670 nm (Figure 2B). Although the color-tuning was not significant, the wavelength shift of 70 nm further demonstrated the influence of polymerization-mediated microenvironment variations on molecular fluorescence [45,46]. A systematic investigation of polymer chain polarity versus emission properties by employing various monomers and fluorophores would be valuable in order to further understand this system.

Recently, we employed a naphthalene diimide (NDI)-derived AIE-active initiator for the ATRP of a series of styrenic monomers [47]. It was found that the polymerization can induce a through-space charge transfer process (TSCT) from the monomeric units (as donors) to the NDI core (as an acceptor). As a result, the emission color of the polymers in the solid state can be tuned from blue to green by increasing the electron-donating ability of the monomers (Figure 2C). Interestingly, an unexpected emission variation was observed due to the chain end group transformation during the polymerization process. The newly formed vinyl benzene end group can induce a structurally remote TSCT to NDI and generate a yellow emission. This end group effect can be engineered in a controlled manner thanks to the living behavior of ATRP, and thus the maximum emission wavelength of NDI-PS film can be shifted from 475 to 528 nm by tuning the transformation degree.

We further used a one-step polymerization approach to achieve end group variations and desired molecular weight at the same time [47]. As a result, the polymer emission color can be tuned from yellow to blue simply by increasing the polymer chain length, with the maximum emission wavelength continuously shifting from 540 nm to 470 nm in polymer films (Figure 2C). The proposed mechanism was validated through theoretical calculations in combination with molecular dynamic (MD) simulation and time-dependent density functional theory (TD-DFT). Clearly, TSCT can be utilized as another efficient tool to manipulate the molecular fluorescence of AIE polymers in solid or aggregated states [48,49], although studies in this direction are still in their infancy.

**Figure 3:**
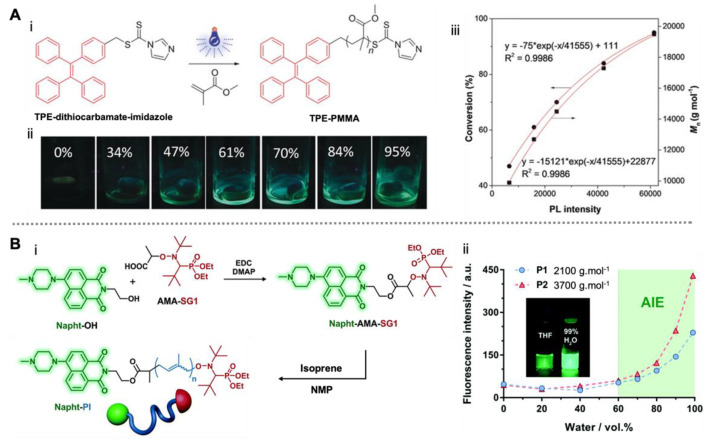
Single-fluorophore AIE polymers prepared by RAFT polymerization and NMP. (**A**) Illustration of RAFT polymerization (i) and photographs of the polymer solutions at different conversions under UV irradiation (ii), as well as the exponential relationship of conversion and *M*_n_ with emission intensity (iii). Reproduced with permission from [57], copyright 2018, John Wiley and Sons, Inc. (**B**) Illustration of NMP synthesis (i) and the AIE activity of the resulting polymers (ii). Reproduced with permission from [60], copyright 2017, Royal Society of Chemistry.

Similarly to ATRP, RAFT polymerization is one of the most frequently used methods for designing well-defined polymeric architectures, which can be conducted for a wide range of monomers under mild reaction conditions [28]. RAFT polymerization has been employed for the efficient synthesis of biocompatible AIE polymers for bioimaging [50,51,52,53] and drug delivery [54,55,56]. In 2018, Tang and co-workers designed a series of tetraphenylethylene (TPE)-based dithiocarbamates for RAFT polymerization [57]. These RAFT agents enabled the visible light-controlled polymerization of various (meth)acrylate monomers. Importantly, the polymerization process could be visualized in situ through the emission variations of TPE (Figure 3A). The TPE-derived RAFT agent was non-emissive in the reaction mixtures, but gradually showed increasing fluorescence intensity along with the growth of TPE polymers, as observed by the naked eye. This is because the TPE fluorophore is sensitive to an increase in local viscosity, which can restrict the molecular motion. Based on this strategy, a superior correlation between the polymer molecular weight and emission intensity was obtained quantitatively (Figure 3A), which provided a convenient approach to monitoring the controlled radical polymerization process without impairing the reaction.

The versatility of this technique was further demonstrated in 12 different polymerization systems [57]. Note that this sensitivity to microenvironment variations is much more controllable in the fluorophore–polymer conjugate system, as the physical blending method was not able to achieve the same efficacy without direct interaction between the fluorophore and the polymer segments [57]. Similar structural sensitivity has been also reported in the RAFT polymerization of TPE-derived monomers, as a shorter spacer between TPE and the polymer backbone results in a stronger restriction of molecular motion, offering a higher fluorescence quantum yield [58]. Recently, AIE fluorophores were also used in self-reporting precipitation polymerization, and the resultant fluorescent polymer nanoparticles exhibited potential in biolabeling and photo-controllable immunotherapy [59]. Therefore, deepening the understanding of the structure–property relationships of well-defined AIE polymers may bring new opportunities for their applications.

Although it has a relatively narrower range of compatible monomers compared to ATRP and RAFT polymerization, NMP exhibits powerful controllability of radical polymerization with simple reaction components [29]. We developed an AIE-active NMP initiator based on naphthalimide-derived alkoxyamine (Napht-AMA-SG1) [60]. The initiator was used for the synthesis of the well-defined single fluorophore polyisoprene (Napht-PI, Figure 3B). Similarly to previous reports, the fluorescence emission of the polymers in their aggregated state was stronger with higher molecular weight. Interestingly, the AIE fluorophore showed a significant blue shift of the maximum emission wavelength from about 520 nm to ~505 nm and ~490 nm, after the resulted polyisoprene grew to 2100 g.mol^−1^ and 3700 g.mol^−1^, respectively (Figure 3B). This phenomenon is likely due to the combined effect of microenvironment variations and charge transfer, which required further investigation for experimental evidence. The single-fluorophore AIE polymers were applied to the labeling of the polymer prodrug nanoparticles [61,62,63] with similar chemical composition for cancer cell imaging. To date, this is the only example of NMP-synthesized AIE polymers.

Note that the AIE dye-initiated controlled radical polymerization has been used as an efficient synthetic strategy to prepare various functional nanosystems, including temperature- or pH-responsive fluorescent nanoassemblies [64,65]. Its use in biomedical applications may be greatly expanded when the structure–property relationship is better elucidated.

## 4. Single-Fluorophore AIE Polymers Prepared by Ring-Opening Polymerization

Distinct from controlled radical polymerization, ROP is a typical polymerization method for the synthesis of biodegradable polymers that are frequently used in biomedical applications [30,66,67,68]. Owing to the efficient synthesis of degradable polyesters and polypeptides with well-defined structures and tunable chain lengths, ROP has been commonly employed to prepare fluorescent materials [69,70,71]. Indeed, all AIE fluorophores that bear active hydroxyl or amine groups usually can be used as functional initiators for ring-opening polymerization, so that single-fluorophore AIE polymers may be easily obtained.

**Figure 4:**
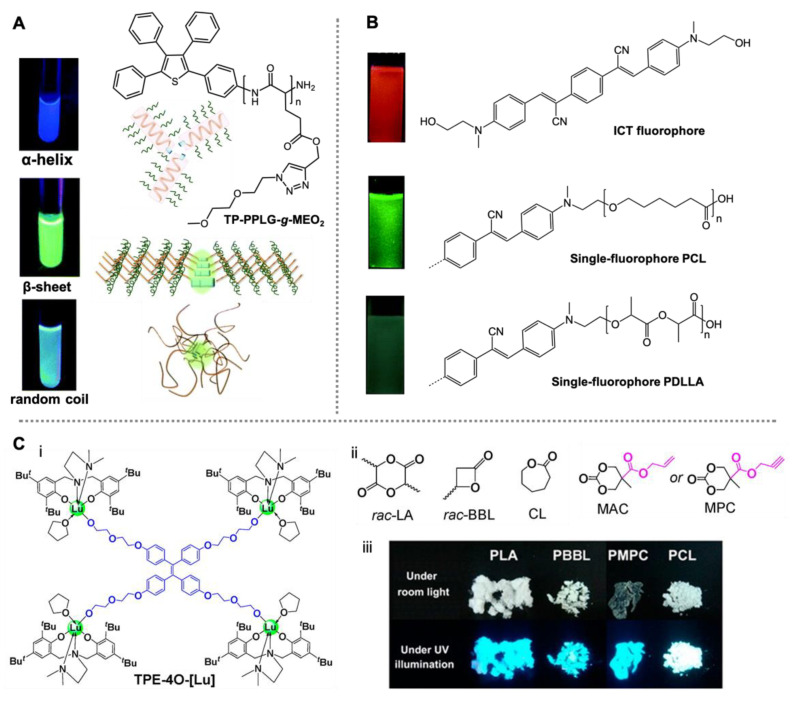
Single-fluorophore AIE polymers prepared by means of ROP. (**A**) Structure and conformation-dependent AIE properties of tetraphenylthiophene-conjugated poly(γ-benzyl-L-glutamate). Reproduced with permission from [73], copyright 2016, Royal Society of Chemistry. (**B**) Structure and polymerization-mediated AIE properties of ICT-based PLA and PCL. Reproduced with permission from [82], copyright 2014, Royal Society of Chemistry. (**C**) Structure of TPE-attached salan lutetium alkoxide complex (i) and the monomers for polymer synthesis (ii), as well as the photographs of the polymer powders under UV irradiation (iii). Reproduced with permission from [86], copyright 2014, American Chemical Society.

In 2012, Hong and co-workers reported the first ROP-synthesized single-fluorophore AIE-active poly(γ-benzyl-L-glutamate) using functionalized tetraphenylthiophene molecules and *N*-carboxyanhydride (NCA) monomers [72]. It was found that the emission intensity of the AIE polymers was highly dependent on the secondary structure of the polypepdtide. The AIE activity is much stronger when the β-sheet conformation is dominant rather than the α-helix, indicated by the higher solid-state quantum yield. Interestingly, the polymers with longer chain lengths have higher fractions of the α-helix structure, and thus lower quantum yields. This is opposite to the molecular weight-dependent emission observed in most AIE polymers. Consequently, the specific polymer conformation has significant impact on the final photophysical properties, including both emission intensity and wavelength (Figure 4A), as also indicated in follow-up studies [73]. This feature can be used to detect variations in the aggregate structure, for example, as a probe for the detection of bovine serum albumin (BSA) [74].

Using a similar ROP method, Kuo and co-workers synthesized a 2,4,6-triphenyl pyridine-functionalized polytyrosine [75]. Although the fluorophore’s emissions were quenched in the aggregated state, the single-fluorophore polymer showed great AIE activity with green emissions at 536 nm, due to the rigid-rod conformation of polytyrosine. To confirm this, they blended the polymer with poly(4-vinylpyridine) and obtained a random coiled complex due to the intermolecular hydrogen bonding, which resulted in a hypsochromic shift to 489 nm with reduced emission intensity. This work again emphasized the importance of polymer conformation in the manipulation of AIE polymer emission. The polymer assembly is considered a significant factor influencing the packing of the fluorophore groups, allowing the modulation of aggregate state fluorescence [76,77]. ROP-synthesized single-fluorophore polypeptides have also been applied to live cell imaging [78].

In addition to polypeptides, ROP-synthesized degradable polyesters represent a pivotal group of polymers that are widely used in drug delivery and medical implants [79,80,81]. In 2013, Yang and co-workers reported the first single-fluorophore AIE-active polylactide (PLA) and polycaprolactone (PCL) using an ICT-based initiator [82]. Although the AIE initiator showed strong red emissions in its aggregate state (581 nm), the resulting PLA and PCL exhibited enhanced fluorescence which, however, shifted to the range of green light (~510 nm) (Figure 4B). Clearly, the enhanced emission should be due to the restriction of molecular motion brought about by the polymer matrix, but the authors did not provide a concrete explanation of the blue-shift phenomenon. This is likely due to the fact that the fluorophore–fluorophore interaction, which is responsible for the red emissions, was inhibited. A similar phenomenon was also observed in other fluorescent polymer systems [83,84,85]. As a result, polymerization can be used as a tool to manipulate the interactions between fluorophore groups and to further tune molecular emissions.

**Figure 5:**
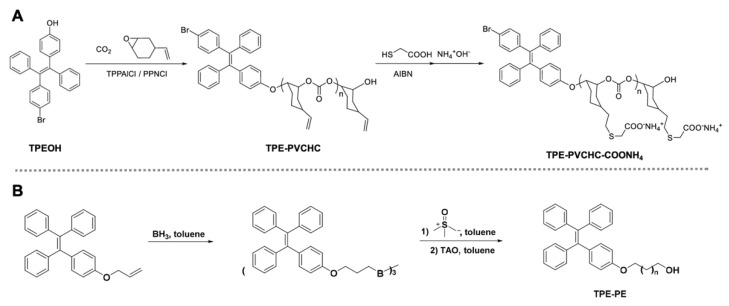
Synthesis of TPE-based polycarbonate (**A**) [88] and PE (**B**) [95].

Thereafter, Cui and co-workers reported a TPE-attached active rare-earth metal catalyst for highly efficient ring-opening polymerizations of cyclic esters [86]. A series of well-defined polyesters with linear, block, or star-shaped topologies were synthesized based on this AIE active complex and various monomers (Figure 4C). Significant AIE activity was observed on the obtained four-arm TPE-attached PLA with strong solid-state emissions. However, the quantitative comparison of TPE initiators and polymers was not provided. Despite this, this work offered a straightforward approach to accessing biodegradable AIE polymers at room temperature [87]. Recently, Wang, Tang and co-workers reported another type of single-TPE-based polycarbonate by means of controllable polymerization of CO_2_ and functional epoxides [88]. The well-defined AIE polymers can be converted to polyelectrolytes with adjustable molecular weight (Figure 5A). Interestingly, the polyelectrolytes showed chain-length-dependent assembly behavior and thus tunable emission intensity. This system was used as a fluorescent probe for the detection of Zn^2+^ ions with molecular-weight-dependent sensing properties. The impact of self-assembly on AIE properties has also been evaluated in other amphiphilic polymer systems [89,90]. For example, Yuan, Wei and co-workers revealed that the fluorescence intensity and fluorescent quantum yield of AIE polymer nanoassemblises increase in the order of vesicles > wormlike micelles > spherical micelles [90].

In biodegradable polyester-based fluorescent systems, it has been shown that the monomer type (e.g., LA or CL) and polymer composition (e.g., copolymerization) have substantial influences on the molecular fluorescence properties [91,92]. Benefiting from the simplicity of ROP-synthesized single-fluorophore polymers, the systematic investigation of their structure–property relationships may provide new opportunities for the design of biocompatible and biodegradable fluorescent materials. This, however, has not yet been achieved.

## 5. Other Well-Defined AIE Polymers 

Although ROP has been the most frequently employed method to synthesize AIE polymers based on polyester and polypeptide, other types of polymers have also been developed, such as polyoxazoline [93] and polyaziridine [94]. These works provide new tools for the study of single-fluorophore polymers. In addition to controlled radical polymerization and ROP, other polymerization techniques have also been selected to design well-defined AIE polymers.

Recenty, Hadjichristidis and Jiang reported [95] the synthesis of a series of TPE-attached polymethylene (equivalent to polyethylene, PE) by means of polyhomologation (Figure 5B), a powerful living polymerization technique involving an ylide (monomer) and an organoborane (initiator) [96]. The resulting TPE polymers can be further functionalized for the synthesis of different types of diblock polymers, including PE-*b*-PS, PE-*b*-PCL and PE-*b*-P*t*BA. It was found that the emission intensity of PE-*b*-P*t*BA in its aggregate state decreased with increasing the chain length of P*t*BA, which is the opposite response to that observed in most AIE polymers. The authors did not give the reason behind this phenomenon, but it could be due to the high flexibility of P*t*BA, which may facilitate the molecular motion of TPE.

Wei and co-authors have employed radical ring-opening polymerization (rROP) to synthesize AIE polymers and prepare fluorescent nanoparticles [97,98]. Although only conventional free radical polymerization was performed in these systems, it should be feasible to obtain well-defined degradable AIE polymers simply by introducing ATRP initiators, NMP nitroxides or RAFT agents [36,99]. Note that rROP has emerged as a new approach for preparing degradable polymeric materials [99]. In addition, ROMP is another type of controlled polymerization based on olefin metathesis [35]. It has also demonstrated great ability in the synthesis of well-defined fluorescent polymers [49], including AIE polymers [100,101,102,103,104] and ladderphanes [105]. Although ROMP-based single-fluorophore AIE polymers have not been reported so far, end-group functionalization may provide an approximate method for this [106,107] if the incorporation of AIE fluorophores can be precisely controlled with high conversion rates.

## 6. Conclusions and Outlook

The last twenty years have witnessed an explosion of research into AIE materials. A number of small molecules, polymers and composites with AIE activity have been developed, some of which have exhibited great potential in optoelectronics and biomedical applications. Compared to AIE small molecules, macromolecular systems—especially well-defined polymers prepared by means of controlled polymerization—have been studied relatively less. To reduce the complexity of AIE polymer systems, the design of single-fluorophore polymers can help in understanding the structure–property relationships in a convenient way thanks to their structural simplicity. Powerful controlled polymerization techniques offer such opportunities to achieve the efficient synthesis of the desired polymer systems. Based on the above discussion, we can summarize the main synthetic strategies and the key factors behind tunable AIE fluorescence as follows.

(i) *Synthesis*. Two major controlled polymerization methods, controlled radical polymerization and ROP, have been employed to synthesize most single-fluorophore AIE polymers. Among various radical polymerization approaches, ATRP and RAFT polymerization are extensively used, owing to their excellent controllability, wide monomer scope and mild reaction conditions. NMP has received less attention, likely due to its less suitable monomers and limited access to the alkoxyamine-based initiator. Despite this fact, NMP can provide superior control of polymer growth with the simplest reaction components (e.g., only initiator and monomers). In the case of ROP, biodegradable polyesters from lactones and polypeptides from *N*-carboxyanhydrides have been considerably reported, with epoxide-based polycarbonate, polyoxazoline and polyaziridine emerging. In addition to these systems, polyhomologation has been used as a unique tool for the synthesis of well-defined single-fluorophore polymethylene and the derived AIE-active block polymers. Notably, all of these methods can realize tunable polymer chain lengths and narrow polydispersity, which are crucial for the investigation of the structure–property relationships of AIE polymers.

(ii) *Mechanism*. As indicated by various single-fluorophore macromolecular systems, the key parameters responsible for tunable emissions include the matrix microenvironment, TSCT, dye–dye interactions and polymer conformation. The variation of the microenvironment is usually induced by choosing monomers with different polarities, which may generate emission wavelength shifts. This can also be achieved by tuning the electronegativity of the monomers if there is a TSCT process between the repeating units and the AIE fluorophore. In the case of TSCT, the fluorescence color can be further manipulated through end-group engineering. On the other hand, if the fluorophore emission is dependent on the packing mode, it can also be tuned by varying the fluorophore concentration, which is feasible by simply controlling polymer chain length. Furthermore, the self-assembly of amphiphilic AIE polymers offers a precise method to modulate the polymer conformation and thus the molecular packing of AIE fluorophores. Subsequently, tunable emission wavelengths and/or intensities could be achieved. Note that in most AIE polymers, the fluorescence intensity increases with the growth of the polymer chains due to the enhanced restriction of molecular motion. Nevertheless, when the polymer chains are highly flexible, the contradictory phenomenon has been observed.

Overall, various single-fluorophore AIE polymeric systems have been explored and certain mechanisms dictating the emission intensity and wavelength have been revealed. However, the systematic investigation of the structure–property relationships of these AIE macromolecules with comprehensive factor analysis remains limited. Further efforts are required to build robust guidelines for the design of well-defined AIE polymers. For example, various single-fluorophore biodegradable polyesters have been synthesized, but a quantitative comparison in regard to emission wavelengths and quantum yield has not been carried out among different monomer types and varied polymer chain lengths. Moreover, the effect of the matrix microenvironment has not been fully elucidated, as the monomer polarity and chain flexibility may interact to disrupt the observed photophysical properties. On the other hand, charge transfer and clusterization-triggered emissions [108] are usually not considered in most systems, which may potentially influence fluorescence. Therefore, some single-fluorophore polymer systems could be worth revisiting with the help of theoretical calculation methods, including TD-DFT and MD simulations, in addition to the design of a new library of AIE polymers. Furthermore, ROMP and rROP represent alternative controlled polymerization techniques for the synthesis of single-fluorophore AIE polymers, in addition to controlled radical polymerization and ROP.

In general, well-defined AIE polymers have shown their great potential in biomedical applications, in particular in nano-assemblies built for fluorescent probes, bioimaging and drug delivery [109,110,111,112,113,114]. Single-fluorophore polymers provide a unique strategy to manipulate molecular fluorescence through precision macromolecular engineering. Although single-fluorophore polymers are indeed not a new concept, the rise of AIE molecules has brought tremendous new vitality to their chemical design. In parallel with the understanding of their structure–property relationships, the question of how to further fabricate efficient stimuli-responsive systems with controllable and significant signal outputs by interfering with one of the four key parameters could provide a promising future research direction, in regard to their multi-tunable fluorescence properties (e.g., emission color). Meanwhile, during this process, machine learning could be a powerful tool to predict the photophysical properties of the designed AIE systems and thus reduce the experimental workload [115,116]. The combination of controlled polymerization, AIE molecular synthesis, theoretical calculation and machine learning could potentially lead to the development of novel fluorescent materials with real-world applications in the near future.
